# *Campylobacter jejuni* in Wild Birds: Current Status and Challenges

**DOI:** 10.3390/vetsci13070649

**Published:** 2026-07-02

**Authors:** Ling Zhang, Zichang Liu, Junjun Du, Xuesong Zhang, Fangzhe Ren, Yuanyue Tang, Xinan Jiao, Jinlin Huang

**Affiliations:** 1 Jiangsu Key Laboratory of Zoonosis, Jiangsu Co-Innovation Center for Prevention and Control of Important Animal Infectious Diseases and Zoonoses, Yangzhou University, Yangzhou 225009, China; dx120240230@stu.yzu.edu.cn (L.Z.); 18035474436@163.com (Z.L.); 18852991697@163.com (J.D.); dz120230045@stu.yzu.edu.cn (X.Z.); fzren@yzu.edu.cn (F.R.); tangyy@yzu.edu.cn (Y.T.); jiao@yzu.edu.cn (X.J.); 2 Key Laboratory of Prevention and Control of Biological Hazard Factors (Animal Origin) for Agrifood Safety and Quality, Ministry of Agriculture of China, Yangzhou 225000, China; 3 Joint International Research Laboratory of Agriculture and Agri-Product Safety, Yangzhou University, Yangzhou 225009, China; 4 College of Life Sciences, Hainan Normal University, Haikou 571158, China

**Keywords:** *Campylobacter jejuni*, wild birds, foodborne infection, transmission

## Abstract

*Campylobacter jejuni* (*C. jejuni*) is a prevalent zoonotic pathogen that poses a significant threat to global public health, causing gastroenteritis with symptoms ranging from mild diarrhea to severe complications, and is widely distributed among various animal hosts, including poultry, livestock, and wild birds. Among these, wild birds, characterized by their exceptional species diversity, high mobility, and long-distance migratory behavior, are capable of disseminating infectious agents across geographic regions and ecological settings. However, wild birds are increasingly recognized as potential carriers, research on *C. jejuni* in these hosts remains nascent and fragmented, and a unified understanding has yet to emerge. Therefore, this review synthesizes current knowledge on the prevalence patterns, key determinants, and genetic and transmission characteristics of wild bird origin *C. jejuni*, aiming to provide a foundation for subsequent in-depth evaluation of the public health significance of *C. jejuni* derived from wild birds.

## Simple Summary

*Campylobacter jejuni* is a common bacterium that causes diarrheal illness in humans. While domestic animals like poultry are known sources, the role of wild birds in spreading this pathogen remains unclear. This review aims to summarize current knowledge on how common *Campylobacter jejuni* is in wild birds, what factors influence its presence, and how the bacterium spreads among bird populations and potentially to livestock and humans. Our synthesis reveals that wild birds, especially migratory species, frequently carry *Campylobacter jejuni* and can disperse it across large geographical areas. Key factors affecting prevalence include bird species, age, season, and migration patterns. The genetic diversity of *Campylobacter jejuni* from wild birds overlaps with strains found in livestock and humans, suggesting cross-species transmission is possible. We conclude that wild birds represent an underappreciated reservoir for *Campylobacter jejuni* and may contribute to foodborne infection cycles. These findings are valuable to society because they highlight the need for integrated surveillance at the wildlife–livestock–human interface and inform public health strategies aimed at reducing campylobacteriosis risks.

## 1. Introduction

*Campylobacter jejuni* (*C. jejuni*) is a prevalent zoonotic pathogen widely distributed in natural environments, with poultry, livestock, wild birds, and other wildlife serving as key reservoirs [1,2]. In humans, *C. jejuni* infection can result in clinical manifestations such as fever, vomiting, cramping abdominal pain, and watery or bloody diarrhea, representing a significant threat to public health [3,4]. Over recent decades, the global incidence of campylobacteriosis has risen steadily, affecting both developed and developing countries [5].

Wild birds, characterized by their exceptional species diversity, constitute one of the most biodiverse vertebrate groups and play a vital role in maintaining ecological balance [6,7]. However, they can harbor a wide range of pathogens, including viruses, bacteria, and parasites, particularly zoonotic agents, thereby posing potential risks to public health [8,9,10]. Bird migration involves large-scale, seasonal movements between established breeding and wintering sites, typically following consistent flyways [11,12]. The extensive geographic distribution, high mobility, and tendency for dense aggregations during migration enable wild birds to facilitate the spread and dissemination of various infectious agents, contributing to the potential emergence of disease outbreaks [13,14].

As important reservoirs of *C. jejuni*, wild birds have not yet been fully understood in terms of the bacterium’s prevalence and its potential for transmission to livestock and humans. This review therefore synthesizes the current scientific literature on *C. jejuni* in wild birds, summarizing the prevalence and associated influencing factors, as well as the genetic evolution and transmission dynamics of avian-origin *C. jejuni*. The objective is to provide a foundation for subsequent in-depth evaluation of the public health significance of *C. jejuni* derived from wild birds.

## 2. Prevalence of *C. jejuni* in Wild Birds

### 2.1. The Prevalence of C. jejuni Among Different Wild Birds

*C. jejuni* has been detected in a wide range of wild bird species, and preliminary statistics indicate its presence in at least 17 avian orders. However, the prevalence varies markedly among different orders, ranging from 2.68% to 64.17%. Among them, Charadriiformes, Passeriformes, and Anseriformes contributed the largest sample sizes and constituted the majority of the monitoring data. According to Table 1, the highest prevalence was found in Gruiformes at 64.17%, followed by Sulidae with a prevalence of 54.55% [15,16,17,18,19]. Several common and widely distributed orders showed moderate prevalence, approximately between 10% and 25% [20,21,22,23,24]. These include Charadriiformes at 24.80%, Pelecaniformes at 23.08%, Strigiformes at 20.00%, Passeriformes at 18.95%, Ciconiiformes at 18.39%, Procellariiformes at 16.28%, Anseriformes at 14.58%, Apodiformes at 13.16%, Columbiformes at 12.46% and Falconiformes at 11.70%. A few orders had prevalence below 10%, namely, Lariformes at 9.73%, Galliformes at 9.38%, Piciformes at 7.14% and Accipitriformes at 6.22%. Sphenisciformes showed the lowest prevalence at 2.68% [21].

**Table 1:** Prevalence of *C. jejuni* in different orders of wild birds.

Order	Number	N. of Positives	Prevalence
Gruiformes	120	77	64.17%
Sulidae	11	6	54.55%
Charadriiformes	3807	944	24.80%
Pelecaniformes	39	9	23.08%
Strigiformes	300	60	20.00%
Passeriformes	8237	1561	18.95%
Ciconiiformes	87	16	18.39%
Procellariiformes	43	7	16.28%
Anseriformes	4498	656	14.58%
Apodiformes	38	5	13.16%
Columbiformes	1148	143	12.46%
Falconiformes	530	62	11.70%
Lariformes	2416	235	9.73%
Galliformes	160	15	9.38%
Piciformes	28	2	7.14%
Accipitriformes	547	34	6.22%
Sphenisciformes	1788	48	2.68%

### 2.2. The Prevalence of C. jejuni in Wild Bird Populations Across Different Regions

**Figure 1:**
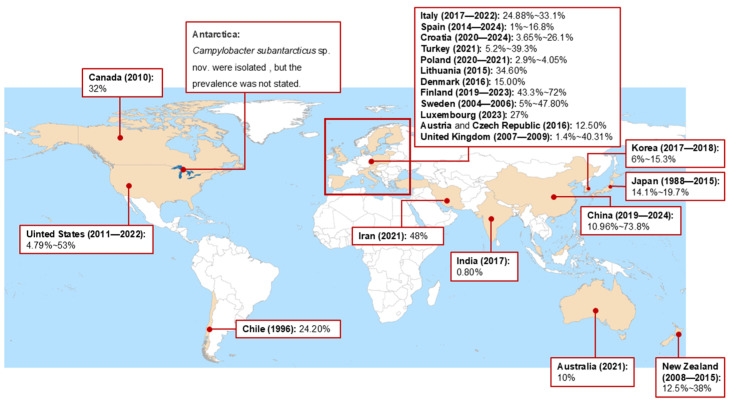
Epidemiology of *C. jejuni* in wild birds. This heatmap shows the prevalence of *C. jejuni* in different countries/regions during various time periods. Countries with no reported prevalence data of *C*. *jejuni* in wild birds available were in white colour, while those with reported data were in yellow. The heatmap was plotted by https://www.bioinformatics.com.cn (last accessed on 4 May 2026).

There is marked geographic variation in the reported prevalence of *C. jejuni* in wild birds across different regions of the world, with economically developed regions generally reporting a greater number of studies and wider prevalence ranges (Figure 1 and Table 2). Notably, *C. jejuni* is the predominant species reported in most regions worldwide and serves as a major pathogen associated with zoonotic diseases.

In Europe, prevalence ranges from 1% in Spain to 72% in Finland, with intermediate rates reported in Italy (24.9%~33.1%), Croatia (3.7%~26.1%), Poland (2.9%~4.1%), Lithuania (34.6%), Luxembourg (27%), the United Kingdom (1.4%~40.3%), Denmark (15%), and Sweden (5%~47.8%) [15,17,18,20,25,26,27,28,29,30,31,32,33,34,35,36,37,38,39,40,41].

In Oceania, New Zealand reports a prevalence of 33% in urban wild birds, while Australia records approximately 10% in Pacific black ducks and Australian wood ducks [24,42]. In South America, Chile shows an overall positive rate of 24.2% in waterfowl, raptors, and urban-adapted birds [43]. In North America, the United States reports a wide range from 4.8% to 53% [22,23,44].

In Asia, China has a prevalence between 11.0% and 19.7%, South Korea between 6% and 15.3%, Japan between 14.1% and 19.7%, and India a notably low rate of 0.8% [16,45,46,47,48,49,50,51].

**Table 2:** Current status and characteristics of *Campylobacter* spp. isolated from various wild birds.

Number	Country	Wild Birds	Sample Type	Method	Positive Rate	Species	References
1	Spain	White Stork, Ring-necked Pigeon, Wood Pigeon, House Sparrow, Crow, etc.	Cloacal swab	Multiplex PCR	16.8% (23/137)	*C. jejuni*, *C. coli*, *C. lari*	[28]
2	Croatia	Yellow-legged Gull	Cloacal swab	PCR, MLST, WGS	14.19% (152/1071)	*C. jejuni, C. lari, C. coli*	[30]
3	Croatia	Yellow-legged Gull, Black-headed Gull, Caspian Gull, Silver Gull, Common Gull	Cloacal swab	Multiplex PCR, MLST	3.65% (64/1753)	*C. lari*	[32]
4	Finland	Ring-necked pheasant	Intestinal samples	PCR, WGS, MLST, cgMLST, wgMLST	72%	*C. jejuni*	[38]
5	Luxembourg	Corvidae, Turdidae, Eurasian Blackcap, pigeon, goose, sparrow	Feces	PCR, MALDI-TOF	27% (97/362)	*C. jejuni*	[18]
6	Italy	Raptors, Omnivorous birds, Gulls	Cloacal swab	Multiplex PCR	24.88% (52/209)	*C. jejuni, C. coli*	[15]
7	Spain	Raptors	Cloacal swab	mPCR	7.5% (52/689)	*C. jejuni*, *C. coli*, *C. lari*	[25]
8	Türkiye	European blackbird, coot, European turtle dove, quail, red-breasted merganser	Cloacal swab	Multiplex qPCR, RFLP-flaA, WGS	32.24% (59/183)	*C. jejuni*, *C. coli*	[19]
9	Poland	Black-headed Gull	Cloacal swab	Multiplex qPCR	4.05% (42/1038)	*C. jejuni*, *C. coli*, *C. lari*	[34]
10	Croatia	Black-headed Gull, Yellow-legged Gull, Caspian Gull, Common Gull, Silver Gull	Cloacal swab	MLST	26.1% (168/643)	*C. jejuni*, *C. coli*, *C. lari*	[31]
11	Poland	Great Tit	Cloacal swab	Multiplex qPCR	2.9% (23/787)	*C. jejuni*	[33]
12	Finland	Western rooks, wild ducks, and pheasants	Fecal and cloacal swabs	MLST, wgMLST, PFGE	43.45% (179/412)	*C. jejuni*	[37]
13	Italy	Raptors	Autopsy intestinal mucosal swab	Multiplex PCR	41.2% (61/148)	*C. jejuni*, *C. coli*,	[27]
14	Denmark	Thrushes and sparrows	Cloacal swab	Biochemical test	15.0% (241/1607)	*C. jejuni*, *C. coli*, *C. lari*, *C. upsaliensis*	[17]
15	Austria, the Czech Republic	Columbiformes, Passeriformes, raptors, Anseriformes, Galliformes, Ciconiiformes	Cloacal swab	MALDI-TOF MS	12.5% (149/1191)	*C. jejuni*, *C. coli*, *C. lari*, *C. helveticus*	[52]
16	Lithuania	Crow, pigeon	Fecal	Multiplex PCR	34.6% (166/480)	*C. jejuni*, *C. coli*	[35]
17	Spain	vulture	Cloacal swab	Biochemical identification	1.0% (1/97)	*C. jejuni*	[29]
18	England	Anseriformes, Falconiformes, Rallidae, Haematopodidae, Charadriiformes, Laridae, Columbiformes, Strigiformes, Turdidae, Paridae, Corvidae, Sturnidae, Passeridae, Fringillidae	Fecal and cloacal swabs	Multiplex PCR, MLST, PFGE	1.4% (29/2084)	*C. jejuni*, *C. coli*, *C. lari*	[20]
19	Britain	Canada geese, grey geese, European starlings	Fecal and cloacal swabs	MLST	40.31% (518/1285)	*C. jejuni*, *C. coli*, *C. lari*	[36]
20	Sweden	Redshank, Black-bellied Plover, Crested Lapwing, Sandpiper, Wood Sandpiper, White-cheeked Pintail	Fecal and cloacal swabs	AFLP, 16S rRNA gene sequencing	47.80% (152/318)	*C. jejuni*, *C. lari* UPTC	[39]
21	Sweden	Charadriiformes, Passeriformes, Raptors	Cloacal swabs	Multiplex PCR, PFGE	89/1794 (5.0%)	*C. jejuni*, *C. coli*	[41]
22	Sweden	Waders, passerines, raptors	Fecal and cloacal swabs	PCR-RFLP	21.6% (388/1794)	*C. jejuni*, *C. coli*, *C. lari*	[40]
23	New Zealand	Ducks, starlings, Canada geese, swans, and Australian waders	Fecal	PCR	33% (583/1768)	*C. jejuni*	[24]
24	Australia	Pacific black duck, Australian wood duck	Fecal	PCR	10% (2/20)	*C. canadensis*	[53]
25	New Zealand	Mallard, European starling	Fecal	PCR, MLST	38%	*C. jejuni*	[54]
26	New Zealand	European starling, house sparrow, welcome swallow, blackbird, Australian magpie, song thrush, New Zealand bellbird, grey-headed canary, goldfinch	Fecal	MLST, PFGE	12.5% (24/192)	*C. jejuni*	[55]
27	South Korea	/	/	MLST	6% (*n* = 1517)	*C. jejuni*, *C. coli*	[46]
28	China	The white-fronted goose, the bean goose, the bar-headed goose, gulls, sandpipers, plovers curlews, and cormorants	Fecal	qPCR	73.8% (329/446)	*C. jejuni*, *C. coli*, *C. lari*, *C. volucris*	[45]
29	Malaysia	Eurasian tree sparrow, jungle myna, white-bellied myna	Cloacal swab	PCR	0	/	[51]
30	Iran	The bald ibis, European starling, house sparrow, red-billed gull, and slender-billed gull	Fecal and cloacal swabs	/	48% (72/150)	*C. jejuni*	[56]
31	China	Crow, rook, mallard, mandarin duck, black swan, rock pigeon	Fecal	qPCR, Multiplex PCR	10.96% (57/520)	*C. jejuni*, *C. coli*, *C. lari*, *C. upsaliensis*	[16]
32	South Korea	Plovers, herons, thrushes, ducks, sandpipers, crows	Fecal and cloacal swabs	Multiplex PCR	15.3% (332/2164)	*C. jejuni*, *C. coli*, *C. lari*	[48]
33	India	Captive wild birds: silver pheasants, white-bellied pheasants, red-crowned cranes, peacocks, ostriches	Fecal	Multiplex PCR	7.84% (8/102)	*C. jejuni*	[57]
34	Japan	Crows, pigeons, tree sparrows	Cloacal swab	16S rDNA	19.7% (34/173)	*C. jejuni*, *C. coli*, *C. fetus*	[49]
35	Japan	Crows, blue magpies, grey starlings, domestic pigeons, white-headed laughingthrushes, oriental turtle doves, tree sparrows, pheasants, bamboo partridges, etc.	Intestinal samples	Biochemical identification	14.1% (44/313)	*C. jejuni*	[50]
36	The United States	American Crow, Fish Crow, Blue Jay, Laughing Gull, Gray-cheeked Thrush, Swainson’s Thrush, Blackbird	Fecal and cloacal swabs	Multiplex PCR, PCR	7.2% (24/333)	*C. jejuni*	[23]
37	The United States	Siskin, Canada goose, brown-headed cowbird, Eurasian collared dove, house sparrow	Fecal and cloacal swabs	PCR	8.0% (3023)	/	[58]
38	The United States	American crows, wild ducks, western scrub jays, wild turkeys in the surrounding cities	Fecal	MALDI-TOF MS, WGS	10.16% (19/187)	*C. jejuni*, *C. coli*	[59]
39	The United States	American crow	Fecal	WGS, GPS	53% (178/337)	*C. jejuni*	[60]
40	The United States	Anseriformes; Charadriiformes, Charadriidae, Laridae	Fecal and cloacal swabs	Multiplex PCR, MLST	9.2% (72/781)	*C. jejuni*, *C. coli*, *C. lari*	[22]
41	The United States	House sparrow, white-throated bunting, rose-breasted grosbeak, blue jay, European starling	Fecal and cloacal swabs	PCR, MLST, PFGE	4.79% (9/188)	*C. jejuni*, *C. coli*	[61]
42	Canada	Seagulls, Canadian geese, ducks	Fecal	qPCR	32%	*C. jejuni*, *C. coli*, *C. lari*	[62]
43	Chile	Waterfowl, Urban-adapted species, Raptors	Cloacal swabs	Lior	24.2% (95/392)	*C. jejuni*, *C. coli*, *C. lari*	[43]
44	/	Adélie penguins, chinstrap penguins, gentoo penguins, giant petrels, black-backed gulls, skuas, snow petrels, king penguins, skuas, snow petrels	Fecal and cloacal swabs	PCR, MALDI-TOF, Whole genome sequencing	6.23% (142/2287)	*C. lari*, *C. subantarcticus*, *C. volucris*, *C. jejuni*, *C. peloridis*	[21]

### 2.3. Prevalence of Other Campylobacter Species in Wild Birds

Although *C. jejuni* is the most frequently reported *Campylobacter* species in wild birds, a diverse range of other species within the genus have also been identified in avian hosts. These include *Campylobacter coli* (*C. coli*), *Campylobacter lari* (*C. lari*), *Campylobacter canadensis* (*C. canadensis*), *Campylobacter subantarcticus* (*C. subantarcticus*), and *Campylobacter volucris* (*C. volucris*) [25,26]. Among these, *C. coli* and *C. lari* are the most frequently detected non-jejuni species and are also recognized as important causes of human gastroenteritis, although they are less prevalent than *C. jejuni* in clinical settings [5,42]. The prevalence of *C. coli* in wild birds varies considerably across studies, with reported rates ranging from 0.3% to 8.2% in European gulls [31,34], 2.7% in migratory waterfowl in China [45], and up to 11.5% in raptors from Italy [27]. Similarly, *C. lari* has been detected at rates of 1.2% to 9.7% in gull populations across Europe [32,34], and has been found in Antarctic penguin species with a prevalence of 4.5% [21]. Notably, some studies have reported that *C. lari* can be the dominant species in specific bird taxa, such as gulls and waders, suggesting potential host adaptation [39,40]. The co-occurrence of multiple *Campylobacter* species in the same wild bird populations raises important questions about interspecies competition, niche differentiation, and the potential for genetic exchange through horizontal gene transfer, which may have implications for the emergence of novel pathogenic strains.

## 3. Determinants of *C. jejuni* Prevalence in Wild Birds

### 3.1. Host-Related Factors

The prevalence of *C. jejuni* in wild birds is not a random occurrence but rather an ecological outcome shaped by the interplay of foraging ecology, migratory behavior, and intrinsic physiological traits. Dietary habits and activity patterns establish the baseline exposure risk, while long-distance migration facilitates pathogen dispersal, and endogenous factors such as body temperature further modulate the likelihood of carriage. Currently, the majority of research findings indicate that the prevalence of *C. jejuni* in carnivorous or omnivorous birds is significantly higher than that observed in herbivorous birds [15,25,40]. For instance, Mencía-Gutiérrez et al. [25] revealed a significantly higher prevalence of *C. jejuni* in carnivorous raptors (9.4%) compared to scavenging or insectivorous raptors (3.1%). In contrast, Hock et al. [18] found that omnivorous birds exhibited a significantly higher prevalence (49%) compared to both carnivorous (11%) and herbivorous (9%) birds. Moreover, Mencía-Gutiérrez et al. [25] noted that the prevalence of *C. jejuni* was significantly greater in nocturnal raptors (15.3%) than in diurnal raptors (6.0%).

Bird migration behavior represents a complex ecological phenomenon and is one of the key factors influencing the prevalence of *C. jejuni* in these birds. A survey conducted in South Korea on the prevalence of *C. jejuni* in wild birds revealed that migratory birds (specifically passage, wintering, and summering birds) exhibited a significantly higher prevalence rate of *C. jejuni* compared to resident birds [48]. In detail, the infection rate among passage birds such as sandpipers and curlews was found to be 19.0% (23/121) [48]. For wintering birds like ducks, the infection rate was 16.7% (286/1712), while summering birds including egrets, herons, and thrushes had an infection rate of 12.3% (18/146) [48]. All these rates were markedly higher than that of resident birds, which stood at only 2.7% [48]. It is important to note that the diverse migratory behaviors exhibited by different populations, including variations in migration routes and distances, can differ significantly among species of migratory birds [63]. These factors, along with food distribution and changes in habitat, may influence the spread and distribution of pathogens, ultimately leading to disparities in the prevalence of *C. jejuni* across various groups [40,45].

In addition, factors such as avian age and body temperature may influence the prevalence of *C. jejuni*. Konicek et al. [52] reported that the prevalence of *C. jejuni* among juvenile birds was 18.1%, which is significantly higher than the 8.47% observed in adult birds. This discrepancy may be attributed to the fact that the immune systems of juvenile birds are not yet fully developed [22,64]. Although wild birds typically sustain a relatively elevated body temperature, the average body temperature of certain larger species, such as barn owls and common vultures, is approximately 39.4 °C. In a study by Casalino et al. [15], no *C. jejuni* was detected in birds with body temperatures below 40 °C or above 42.2 °C. Consistent with this observation, the average body temperature of certain larger species, such as barn owls and griffon vultures, falls below this range, and no *C. jejuni* was isolated from these birds [15]. While this observation is intriguing, the in vitro growth range of *C. jejuni* is relatively broad (approximately 30~47 °C), and since the body temperatures of most bird species fall within this range, temperature alone is unlikely to be the primary determinant of colonization. Furthermore, direct evidence linking host body temperature to carriage rates in wild birds remains limited, and other ecological or physiological factors, such as foraging ecology, migratory behavior, immune competence, and environmental exposure, likely play more prominent roles [2,15,25,40,45]. Thus, while body temperature cannot be excluded as a contributing factor, its significance as a primary determinant requires further investigation.

### 3.2. Geographic Factors

Both seasonal temperature regimes and habitat characteristics exert a measurable influence on *C. jejuni* prevalence in wild avian populations, with warmer periods and rural or contaminated environments consistently associated with elevated carriage rates. Mohan et al. [24] indicates that the occurrence of *C. jejuni* in wild birds is notably higher during warmer months, specifically in September, October, November, and January. Similarly, Hald et al. [17] observed a significantly higher positivity rate in summer (20.0%, 180/901) compared to winter (15.9%, 112/706).

Habitat-mediated effects are equally pronounced. In a study of pathogen occurrence in wild birds at landfill sites in Iran, Malekian et al. [56] suggested that the highly contaminated environment of landfills may contribute to elevated infection rates in gulls, starlings, and crows. Conversely, Tryjanowski et al. [33] observed that rural great tits exhibited *C. jejuni* colonization odds more than 2.5 times higher than those of their urban conspecifics, with positivity increasing in proximity to livestock operations. Moreover, Mourkas et al. [65] demonstrated that urban-adapted species such as crows and starlings harbor greater *C. jejuni* genotypic diversity, suggesting that anthropogenic landscapes may alter transmission dynamics and strain circulation patterns.

### 3.3. Anthropogenic and Methodological Factors

Methodological variation constitutes a significant, non-biological source of disparity in reported *C. jejuni* detection rates among wild birds, with sample freshness, transport conditions, and the analytical technique employed each exerting measurable influence on recovery outcomes. Regarding sample integrity, French et al. [55] documented a substantially higher detection rate in fresh fecal specimens (15.2%) than in dry specimens (6.7%), underscoring the sensitivity of *C. jejuni* to desiccation. The detection rate in fresh feces was significantly higher than that observed in dry feces.

Transport conditions also critically influence recovery rates; maintaining low temperatures (e.g., 4 °C) and minimizing transport duration significantly prolong the viability of *C. jejuni* [66]. In terms of diagnostic methodology, quantitative PCR (qPCR) yields a higher detection frequency (32%) relative to conventional culture (29%) in waterfowl, as reported by Van Dyke et al. [62]. This differential is attributable to the capacity of qPCR to identify viable but non-culturable (VBNC) cells and extracellular DNA, whereas culture-based approaches are constrained by the selectivity and recovery efficiency of enrichment media [67,68,69].

## 4. Public Health Implications of *C. jejuni* in Wild Birds

### 4.1. Genomic Diversity of C. jejuni in Wild Birds

Although wild bird-derived *C. jejuni* exhibits high overall genomic diversity, its core genomic backbone remains highly conserved throughout evolution. Wild bird-derived *C. jejuni* consistently retains the typical genomic background features of the species. Its genome is a circular DNA molecule approximately 1.6~1.8 Mbp in size, with a GC content of around 30%. The genome sizes of eight *C. jejuni* strains obtained from wild birds in Japan ranged from 1.65 to 1.77 Mbp, falling within the typical range observed for the species when compared with isolates from other sources [46,70].

*C. jejuni* in wild birds exhibits a distinct population structure that is closely associated with host taxonomy, demonstrating a high degree of host specificity. As natural reservoirs with high species diversity, wild birds encompass a wide range of gut environments across taxa such as Anseriformes and Passeriformes, which may promote the emergence of host-adapted subpopulations of *C. jejuni* [2]. Moreover, the global migratory behavior of birds may facilitate the cross-regional dissemination of bacterial strains, thereby increasing opportunities for genetic exchange [71,72,73]. Consequently, numerous studies have compared the genetic relatedness of *C. jejuni* isolates from wild birds with those from poultry, humans, and other animals [2,74,75]. Some research indicates that the population structure of *C. jejuni* in wild birds is closely associated with host taxonomy, suggesting a high degree of host specificity [2]. Griekspoor et al. [2] conducted an analysis of *C. jejuni* isolates obtained from starlings in Sweden, Australia, and the Azores using MLST. Their findings revealed that starling isolates, specifically ST-1324 and ST-1342, formed distinct independent clusters. This indicates a significant level of host specificity and highlights their marked differences from isolates derived from poultry or humans. Similarly, Kovanen et al. [37] also discovered that the ST of *C. jejuni* isolates obtained from rooks and wild ducks, such as ST-1282 and ST-6460, were predominantly distinct from those previously identified in humans and other animals.

The sequence type repertoire of wild bird-derived *C. jejuni* is exceptionally rich, containing a large number of newly identified sequence types and alleles, and its genetic diversity is significantly higher than that of isolates derived from poultry and humans (Table A1). The high degree of genotypic variation among wild bird isolates has been documented in numerous regional surveys [16,18,76]. A study in South Korea isolated 60 *C. jejuni* strains from 12 wild bird species and identified 32 STs through multilocus sequence typing (MLST) analysis [46]. Similarly, Ječmenica et al. [76] applied MLST to 106 *C. jejuni* isolates from yellow-legged gulls, revealing 44 STs, including 11 novel types. In a study of wild birds conducted in the Beijing region of China, MLST analysis of 36 *C. jejuni* isolates assigned them to four clonal complexes together with several sequence types that could not be grouped into any defined complex; among these, 20 STs (accounting for 55.56% of the total) and 6 alleles were documented for the first time [16]. In another study, Mäesaar et al. [77] conducted MLST on 101 *C. jejuni* isolates derived from wild birds and identified 68 STs, representing the highest genetic diversity compared to isolates from other sources such as humans, poultry, and cattle.

### 4.2. Transmission Dynamics of C. jejuni in Wild Birds

As summarized in Table A1, wild birds constitute a significant reservoir for *C. jejuni*; accumulating evidence indicates that avian-derived strains can be transmitted across species boundaries to poultry, livestock, and human populations via multiple environmental pathways. Genomic evidence supports the potential for direct zoonotic transmission. *C. jejuni* derived from wild birds can infect humans, poultry, and livestock directly or indirectly through fecal contamination [18,46,78]. For example, French et al. [55] revealed that the PFGE profiles of ST-45 isolates obtained from wild bird feces in a city park playground in New Zealand were indistinguishable from those of isolates derived from human cases within the same country. This finding suggests that feces from wild birds may be a potential source of campylobacteriosis among preschool children. Mohan et al. [54] likewise reported ST-45 and ST-177 in mallards and starlings, sequence types that have been associated with human infections. Furthermore, wild bird-derived *C. jejuni* strains belonging to ST-677, ST-45, and ST-267 have shown genotype overlap with poultry isolates, and ST-806 has been linked to livestock abortion cases [54,79]. Collectively, these molecular overlaps highlight the potential for cross-host transmission facilitated by wild birds. Domestic poultry, particularly free-range flocks, may become infected through direct contact with wild birds or their excreta. Kovanen et al. [37] reported higher *C. jejuni* positivity rates in domestic ducks that cohabited with wild waterfowl. However, a large-scale UK study involving 2084 wild birds indicated that the predominant direction of transmission may be from poultry to wild birds [20]. In this study, both poultry-associated and wild bird-specific sequence types were isolated from wild birds, whereas no wild bird-specific strains were detected in commercial poultry populations.

Wild birds are recognized as a major source of *C. jejuni* contamination in surface waters. A Canadian study comparing *C. jejuni* isolates from river water and waterfowl revealed 100% genetic homology, suggesting that waterfowl contribute significantly to aquatic pollution [62]. Human campylobacteriosis outbreaks in Norway in 1994 and 1995 were suspected to be linked to drinking water contaminated by feces from bean geese [80]. The hydrological cycle plays a critical role in the dissemination of *C. jejuni* among wild birds, poultry, livestock, and humans. *C. jejuni* can enter surface water through wastewater discharge, and animals such as cattle, chickens, and wild birds may become infected via contact with contaminated water. Subsequently, their feces can contaminate agricultural land, serving as a persistent source of infection for other animals, food products, and water supplies [81,82].

*C. jejuni* from wild birds can also be transmitted to humans and livestock through soil and crop contamination. During an outbreak of human campylobacteriosis in Alaska, USA, linked to the consumption of raw peas, *C. jejuni* isolates from patients, environmental samples, and wild sandhill cranes displayed indistinguishable PFGE patterns. It was hypothesized that sandhill cranes defecated in pea fields, leading to contamination, and that ingestion of raw, contaminated peas constituted the primary transmission route [83].

Additionally, insects such as flies may act as mechanical vectors in the transmission of *C. jejuni* among wild birds. In a study by Hock et al. [18], *C. jejuni* isolates ST-1044 and ST-475, which were identical to those found in human clinical cases, were detected in swifts. Given that swifts feed on flying insects, it has been speculated that insects may play a role in the transmission cycle of *C. jejuni* to wild birds. Collectively, these findings underscore the interconnectedness of wild bird ecology, environmental contamination, and public health risk.

### 4.3. Virulence Factors and Antimicrobial Resistance of C. jejuni in Wild Birds

Available data indicate that *C. jejuni* isolates from wild bird hosts routinely possess a defined suite of virulence genes associated with adherence, invasion, and cytotoxicity, although natural infections seldom produce observable clinical disease [84,85]. Among the most frequently detected determinants are the cytolethal distending toxin gene cluster, which comprises *cdtA*, *cdtB*, and *cdtC*, along with flagellin genes (*flaA*, *flaB*), the invasion-associated gene *ciaB*, and the fibronectin-binding adhesin gene *cadF* [16,49]. The presence of *cadF* has been documented in wild bird populations across multiple countries, including China, Poland, and Japan [16,26,49].

Beyond the carriage of virulence genes, the antimicrobial resistance profile of *C. jejuni* derived from wild birds presents additional dimensions of public health concern. Overall, the antimicrobial resistance profile of *C. jejuni* derived from wild birds is characterized by a comparatively high degree of baseline susceptibility, widespread resistance to certain antimicrobial agents, and the incipient emergence of multidrug resistance. Compared with isolates originating from intensively reared poultry, wild bird strains generally exhibit lower rates of antimicrobial resistance and multidrug resistance [74,86]. However, this does not imply that the associated public health risk can be disregarded. Multiple studies have consistently demonstrated that resistance to quinolones (e.g., ciprofloxacin, nalidixic acid, enrofloxacin) and sulfonamides is the most prevalent among wild bird isolates, with fluoroquinolone resistance having already become a global phenomenon [74,86]. Multidrug-resistant strains have been detected in wild birds sampled at a wildlife rescue centre in Italy, indicating a progressive accumulation of resistance traits in wild hosts [87]. With respect to resistance mechanisms, strains from wild birds share key resistance determinants with isolates from poultry and human clinical cases (Table A1). Nearly all wild bird-derived strains carry *OXA*-type β-lactamase genes, which confer an intrinsic basis for resistance to β-lactam antibiotics [74,86,88,89]. High-level quinolone resistance is predominantly mediated by the T86I point mutation in *gyrA*, a mutation that has been detected in isolates from wild birds alike [74,86].

## 5. Future Perspective

Wild birds serve as important natural reservoirs for *C. jejuni* [18]. Their migratory behavior facilitates the transmission and spread of this pathogen, effectively acting as a mobile source of infection that connects livestock, humans, and natural environments [45,89]. Therefore, in-depth research on *C. jejuni* in wild birds, particularly its transmission pathways, is of great significance for early warning of emerging risks.

Current studies primarily focus on the detection of *C. jejuni*, with some involving antimicrobial resistance analysis and comparisons of population structure across hosts [40,74,90]. However, several limitations remain apparent [22,45,87]. Most existing surveys are situational studies targeting specific regions or bird species, lacking systematic, long-term monitoring networks across ecological zones. This makes it difficult to reveal macro-level epidemiological patterns. Moreover, research methods remain relatively conventional, relying heavily on bacterial culture and typing, while underexploring genetic evolutionary characteristics, virulence factors, and resistance mechanisms of bacterial strains [2,90,91]. As a result, effective traceability and accurate assessment of their true pathogenic risks are hindered.

Finally, although numerous studies have reported carriage rates in wild birds, direct evidence and quantitative models are still lacking regarding their specific role in transmission dynamics (such as how strains are disseminated to farms or water bodies) and how environmental pressures (e.g., climate change, habitat destruction) influence bacterial shedding patterns. Moreover, a critical limitation of current molecular epidemiological studies is that the detection of shared genotypes or antimicrobial resistance determinants between wild bird, livestock, and human isolates has often been taken as suggestive of cross-host transmission. However, such overlaps alone do not constitute definitive evidence of transmission directionality or frequency [20]. The epidemiological significance of these shared genetic markers remains difficult to interpret without integrating source-attribution models and population genetic frameworks that can distinguish between occasional spillover events and sustained transmission cycles [38,65]. Beyond molecular approaches, future investigations should also incorporate field epidemiological tools, such as GPS tracking of individual birds, to directly link host movement patterns with bacterial dispersal and environmental contamination risks. Combining high-resolution genomic data (e.g., whole-genome sequencing and core-genome MLST) with real-time spatial–behavioral data would provide a powerful framework for elucidating the true extent of *C. jejuni* transmission among wild bird populations, livestock, and humans, and for assessing the impact of anthropogenic and climatic changes on these dynamics.

Looking forward, advanced technologies such as whole-genome sequencing should be utilized to thoroughly analyze the genetic background of *C. jejuni* isolated from wild birds. Through comparative genomics, evolutionary relationships between wild bird isolates and strains from human clinical cases and livestock can be clarified, aiding in tracing the origin of outbreaks. Simultaneously, research on pathogen–host interactions should be strengthened to explore how the immune physiology of different bird species influences their carrier status. From a prevention and control perspective, it is essential to implement comprehensive surveillance programs and establish interdisciplinary, cross-regional collaborative networks for long-term and continuous sampling and monitoring of wild bird populations, particularly along major migration routes. In addition, integrating ecology, bird banding data, and microbial genomics will help map the spatiotemporal dynamics of *C. jejuni* in wild bird populations, with special attention to species that have frequent contact with humans and livestock.

## Data Availability

No new data were created or analyzed in this study. Data sharing is not applicable to this article.

## Appendix A

**Table A1:** Comparison of *C. jejuni* Molecular Characteristics across Wild Bird, Livestock, and Human Hosts.

Number	Source of Isolate	Dominant STs/Clonal Complexes	Virulence-Associated Genes	Antimicrobial Resistance (AMR) Profile	References
1	Wild birds	ST-1324, ST-1342, ST-1264, CC-1347, ST-1020, ST-177, CC-45, CC-21, CC-283	Not investigated	Not investigated	[2]
	Livestock (Goose, Chicken, Cattle, Sheep)	CC-21, CC-45, CC-283	Not investigated	Not investigated	
	Human Clinical	CC-21, CC-45, CC-283	Not investigated	Not investigated	
2	Wild birds	CC-692, CC-52, CC-952, CC-1275; ST-995, ST-991, ST-2367, ST-52, ST-692, ST-1540	*flaA* (100%), *cadF* (100%), *cdt* gene cluster (100%); truncated *cdt* cluster in crow, jackdaw, and silver pheasant	Streptomycin (36.8%), tetracycline (29.8%), gentamicin (29.8%); MDR 33.3%	[16]
	Livestock (Poultry/Cattle)	ST-995, ST-991, ST-2367, ST-52, ST-692, ST-1540	Not investigated	Not investigated	
	Human Clinical	ST-995, ST-991, ST-2367, ST-52, ST-448, ST-951	Not investigated	Not investigated	
3	Wild birds	Jackdaw: ST-1282, ST-6460, ST-4565, ST-7841 (generalist clade); Mallard: ST-2314, ST-1299, ST-2839, ST-991, ST-995; Pheasant: ST-19	Novel *cdtABC* gene cluster in 59% jackdaws and 1 mallard duck; T6SS in 49% jackdaws and 72% mallard ducks; *cdtABC* degenerated in most jackdaws and mallard ducks; intact *cdtABC* in pheasants	Pheasant: 100% *gyrA* T86I, *blaOXA-61*; Jackdaw: 14% *tetO*, 1 isolate *gyrA* T86I, *blaOXA-184*; Mallard: 3% *tetO*, *blaOXA-184*	[37]
	Livestock (Poultry/Cattle)	ST-6460, ST-19	Not investigated	Ciprofloxacin resistance common	
	Human Clinical	ST-2314, ST-19	Not investigated	Ciprofloxacin resistance common	
4	Wild birds	Duck: CC-1034 (28%), CC-45 (19%), CC-692 (9%); Starling: CC-45 (28%), CC-177 (9%); ST-1324, ST-1342, ST-137, ST-2378, ST-3961, ST-45, ST-53, ST-583, ST-692, ST-991, ST-992	*flaA* SVR and *porA* genotyping: greater *flaA* diversity in ducks; *flaA* alleles 1170, 1219, 1221, 1222, 1235, 16, 209, 213, 219, 520, 56, 69, 73, 787, 85, 89 found only in ducks; *porA* allele 6 predominant in ducks	Not investigated	[54]
	Livestock (Poultry/Cattle)	ST-45	Not investigated	Not investigated	
	Human Clinical	ST-45, ST-137, ST-583, ST-677, ST-42, ST-53, ST-1255, ST-696, ST-2026, ST-526	Not investigated	Not investigated	
5	Wild birds	ST-1275 CC (43%)–gull-adapted; ST-21 CC, ST-45 CC;	Not investigated	Not investigated	[76]
	Livestock (Poultry/Cattle)	ST-21 CC, ST-45 CC	Not investigated	Not investigated	
	Human Clinical	ST-21 CC, ST-45 CC, ST-828 CC	Not investigated	Not investigated	
6	Wild birds	68 STs (most diverse); ST-4447, ST-6424, ST-220; 53.5% singleton STs; no ST overlap with poultry/cattle/humans (PSI = 0)	Not investigated	Not investigated	[77]
	Livestock (Poultry/Cattle)	Poultry: 53 STs; ST-5 (15.8%), ST-464 (9.4%), ST-6410 (7.9%); Cattle: 17 STs; ST-21 (37.5%), ST-3098 (10.4%)	Not investigated	Not investigated	
	Human Clinical	49 STs; ST-5 (17.4%), ST-50 (12.1%), ST-51 (6.1%)	Not investigated	Not investigated	
7	Wild birds	Blackbird-specific: ST-1229, ST-1260, ST-1303, ST-1342 (~40%); ST-1324 and ST-1342 shared between Sweden and Australia; ST-3576 (Azores) closely related to ST-1249 (Sweden); minimal genetic differentiation despite ~10,000 generations of isolation	Not investigated	Not investigated	[79]
	Livestock (Poultry/Cattle)	ST-267, ST-137 (occasional overlap; cluster with chicken/human genotypes in ClonalFrame)	Not investigated	Not investigated	
	Human Clinical	Rare overlap with wild birds; majority of Blackbird genotypes are host-specific	Not investigated	Not investigated	
8	Wild birds	CC-21, CC-45 (generalist lineages); wild bird-associated genotypes also present	Not investigated	Lowest resistance: CIP (13.43%), NAL (10.45%), TET (19.40%), ERY (7.46%), STR (10.45%), GEN (2.99%); MDR observed (up to 5–6 classes); Genes: *tet(O)* (14.92%), *gyrA* T86I (44.44%), *blaOXA-184* (82.08%), *blaOXA-61* (17.14%), *cmeABC* (100%); Key finding: *blaOXA-184* strongly associated with wild birds	[86]
	Livestock (Poultry/Cattle)	CC-21, CC-45 (most common)	Not investigated	Highest resistance: CIP (85.55%), NAL (75.48%), TET (67.87%), ERY (2.28%), STR (2.85%), GEN (0.19%); MDR: 47.90%; Genes: *tet(O)* (74.33%), *gyrA* T86I (96.22%), *blaOXA-61* (62.14%), *blaOXA-184* (31.56%), *cmeABC* (100%)	
	Human Clinical	CC-21, CC-45 (most common)	Not investigated	High resistance: CIP (76.47%), NAL (74.51%), TET (49.02%), ERY (7.84%), STR (1.96%), GEN (1.96%); MDR: 17.76%; Genes: tet(O) (56.86%), gyrA T86I (97.43%), blaOXA-61 (70.59%), blaOXA-184 (11.76%), cmeABC (100%)	
9	Wild birds	CC1034 (100% MDR), CC354, CC403, CC443, CC446	Not investigated	95% any resistance; CIP (87.1%), TET (8.9%), AXO (46.5%), ERY (1.0%), GEN (0%); MDR 7.9%; CIP + AXO (30.7%), CIP (47.5%) dominant; CC1034 100% MDR	[88]
	Livestock (Poultry/Cattle)	Poultry: ST-464 (CC464), ST-5 (CC353), ST-6410 (CC464); Cattle: ST-21 (CC21) dominant; CC21, CC353, CC179 dominant overall	Not investigated	Poultry: 100% any resistance; CIP (100%), TET (64.3%), AXO (71.4%), ERY (0%), GEN (0%); MDR 42.9% (TET + CIP + AXO). Cattle: 100% any resistance; AXO (100%), CIP (90.2%), TET (85.4%), ERY (0%), GEN (0%); MDR 80.5% (TET + CIP + AXO); ST-21 100% MDR	
	Human Clinical	ST-5 (CC353), ST-50 (CC21) predominant; CC21, CC353, CC179 dominant	Not investigated	94.2% any resistance; CIP (88.1%), TET (14.9%), AXO (45.5%), ERY (2.0%), GEN (0%); MDR 7.9%; CIP + AXO (33.7%), CIP (32.7%) dominant	
